# Human health risk associated with metal exposure at Agbogbloshie e-waste site and the surrounding neighbourhood in Accra, Ghana

**DOI:** 10.1007/s10653-023-01503-0

**Published:** 2023-02-28

**Authors:** Matt Dodd, Lydia Otoo Amponsah, Stephen Grundy, Godfred Darko

**Affiliations:** 1 School of Environment and Sustainability, Royal Roads University, Victoria, BC Canada; 2 Department of Chemistry, Kwame Nkrumah University of Science and Technology, Kumasi, Ghana

**Keywords:** Toxic metal, Agbogbloshie, Urban topsoil pollution, Bioaccessibility

## Abstract

Agbogbloshie in Accra, Ghana, was a center for informal e-waste recycling until it was closed recently. This study investigated the potential health risks of toxic metals (including As, Cd, Cu, Ni, Pb, Sb, and Zn) found in the surface soils based on their concentrations and in vitro bioaccessibility. Mean concentrations at the burning sites were As: 218; Cd: 65; Cr: 182; Cu: 15,841; Ni: 145; Pb: 6,106; Sb: 552; and Zn: 16,065 mg/kg while the dismantling sites had mean concentrations of As: 23; Cd: 38; Cr: 342; Cu: 3239; Ni: 96; Pb: 681; Sb: 104; and Zn: 1658 mg/kg. The findings confirmed the enrichment of potentially toxic metals at the dismantling and burning sites, exceeding international environmental soil quality guidelines. Based on the total metal concentrations, bioaccessibility, and calculated risk indices, the risks associated with incidental ingestion of soil-borne metal contaminants at the dismantling and burning sites were very high. Despite evidence of higher metal concentrations in the communities near the burning and dismantling sites, the human health risk associated with soil ingestion was significantly lower in the surrounding neighborhood.

## Introduction

Electronic and electrical waste (e-waste), such as discarded televisions, computers, laptops, portable phones, and refrigerators, is the fastest-growing domestic waste stream in the world (Amponsah, Sørensen, et al., 2022a, b; Shittu et al., 2021). This is attributed mainly to the high use of electronic and electrical equipment that either generally becomes obsolete in a few years or are not easily repairable (Forti et al., 2020). According to the Global E-Waste Monitor (2020), of the 53.6 million metric tons of e-waste generated in 2019, only 17.4% was collected and recycled properly (Forti et al., 2020). A portion of the unaccounted-for waste ends up in the informal recycling stream. Electrical and electronic equipment contain various metals and metalloids that were used in their manufacture. Informal recycling involves the mechanical dismantling of the equipment to reclaim valuable visible metal components (e.g., Al, Cu and Fe) followed by open burning or acidic leaching to recover the remaining invisible metals in the dismantled pieces (e.g., Ag, Al, Au, Cu, Pd) (Forti et al., 2020). Mechanical dismantling, open burning and disposal of leachate can potentially introduce various metals and metalloids including As, Cd, Co, Cr, Cu, Ni, Sb, and Zn into the environment (Ackah, 2019; Adesokan et al., 2016; Amponsah et al., 2022a, b; Cao et al., 2020; Han et al., 2019; Ouabo et al., 2019; Singh et al., 2018).

Until recently Agbogbloshie, a suburb in Accra, Ghana was used as an informal e-waste recycling site. Prior to their relocation in 2021, the population of the study area was approximately 30,000 (Amoyaw-Osei & Agyekum, 2011). Agbogbloshie is situated near the Odaw River less than 1 km from the central business district of Accra. It was used as a scrap yard for e-waste and other materials including obsolete computers, monitors, refrigerators, television sets, used batteries, vehicles, and sheathed cables (Ackah, 2019). These wastes were manually dismantled at numerous small workshops scattered across the site to retrieve recoverable materials such as copper cables and precious metals. Printed circuit boards, capacitors, sheathed cables, and tires were openly burnt at designated burning sites often resulting in thick dark smoke. These activities resulted in the contamination of soils at the burning areas and dismantling sites. Earlier studies at the burning areas showed elevated levels of metals and metalloids (Vaccari et al., 2019). Other studies (Ackah, 2019; Cao et al., 2020) also confirmed soils in burning areas contain metals such as As, Cd, Cu, Pb, and Zn at levels that far exceeded international environmental soil quality guidelines such as the Dutch Target and Intervention Values (VROM, 2000) and the Canadian Council of Ministers of the Environment (CCME) guidelines (CCME, 2007).

Humans are exposed to the metal and metalloid contaminants incorporated into soils through either ingestion or inhalation of the contaminated soils or dermal contact with the soils (Darko, Dodd, Nkansah, Aduse-Poku, et al., 2017a, b). The human health effects resulting from exposure are metal-dependent. For example, inorganic arsenic is a known human carcinogen and chronic exposure can cause a decrease in the production of red and white blood cells, abnormal heart rhythm and damage to blood vessels while inorganic lead is a probable human carcinogen that affects every organ system (US-EPA, 2002). Although copper and zinc are essential elements, exposure to high doses of copper or zinc can result in gastrointestinal irritation (ATSDR, 2014, 2022; Fosmire, 1990; Taylor et al., 2020).

In human health risk assessments of metal contaminants in soils, oral ingestion is the predominant mode of exposure for the general population albeit workers who dismantle and burn e-waste may also be exposed through the inhalation of smoke and dust particles as well as dermal contact. In vitro bioaccessibility (IVBA) which is the fraction of the total amount of the contaminant in a soil sample that dissolves in a simulated gastrointestinal fluid (US-EPA 2007a) provides a more realistic exposure scenario for the human health risk assessments (Basta & Juhasz, 2014; Health Canada, 2017).

**Fig. 1:**
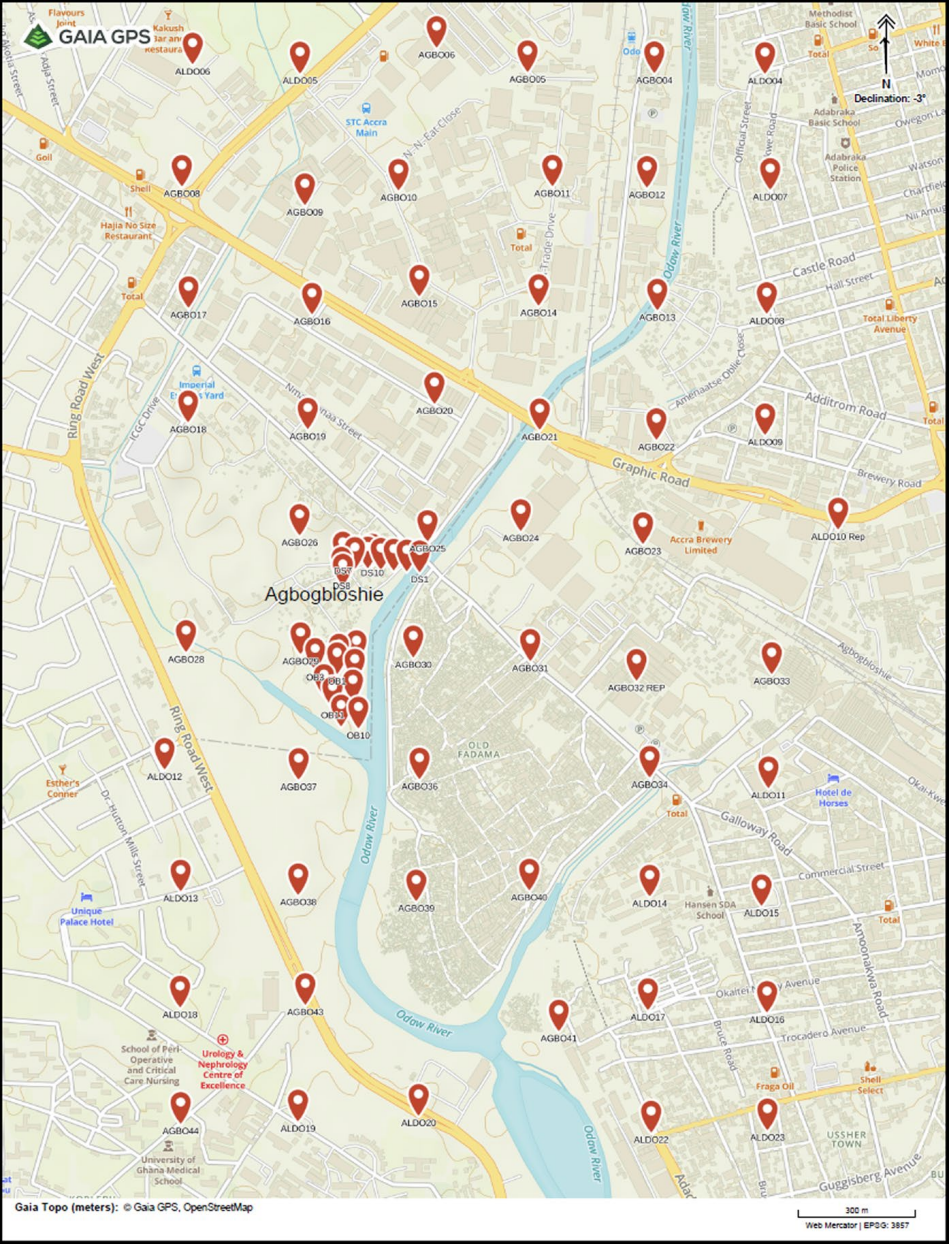
Map of study area showing sampling locations

Agbogbloshie (5°32′45.5"N; 0°13′27.2"W) is surrounded by several localities comprising Kaneshie, South Industrial Area, Abossey Okai, Kokomlele, Ussher Town and Korle Bu (Fig. 1) with various infrastructures such as residences, warehouses, company manufacturing sites, marketplace places, automobile repair shops, carpentry shops and a hospital. Although the presence of metals and metalloids at elevated concentrations at the burning and dismantling areas in Agbogbloshie that pose human health risks has been reported (Ackah, 2019; Cao et al., 2020; Vaccari et al., 2019), there is a lack of data on metal distribution in the surrounding neighborhood. This study was therefore conducted to determine the spatial distribution of metals and metalloids in surface soils in the vicinities of Agbogbloshie. The human health risk based on ingestion of these soil-borne metal contaminants at the dismantling areas, the burning sites and the surrounding neighborhood was determined by incorporating the concentrations of these metals and their in vitro bioaccessibility.

## Methodology

### Sample collection

Surface soil samples were collected at the dismantling sites (*n* = 11), burning areas (*n* = 14), and in the surrounding community (*n* = 64) in December 2020. Sampling points at the dismantling sites were along a linear grid while a judgmental approach based on the accessibility of soil was used to select locations at the dismantling site due to the debris scattered in the area. A global positioning system application (GPS Essentials, Malaga, Spain) was used to design a 300 m by 300 m grid for the surrounding community followed by sampling at the intersects. Sampling locations were shifted to the nearest accessible points for intersects with infrastructure (e.g., buildings, roads) that prevented surface soil sampling. A plastic scoop was used to collect the soil sample from the top 10 cm. The sampling locations are shown in Fig. 1. For background purposes, 12 samples were collected from the Legon Botanical Gardens, located about 18 km away from the Agbogbloshie scrapyard but has similar geological regimes. The Legon Botanical Gardens is a state botanical garden managed by the University of Ghana. Management includes conservation programs that place restrictions on patronage, and they are therefore considered free from human activities which may introduce heavy metals into the soil.

### Sample preparation and analysis

Soil samples were air-dried at ambient temperatures and sieved using 250 µm polyethene sieves.

#### Metals in soil

The sieved soil samples were analyzed for metals using a Niton XL3t GOLDD + X-ray fluorescence (XRF) analyzer based on US-EPA Method 6200 (US-EPA, 2007b). The detection limits for the XRF analysis are included in Table 1. A certified standard reference material (NIST 2711a) was analyzed in triplicate for quality assurance. Thirteen of the samples (representing 13%) were analyzed by inductively coupled plasma-mass spectrometry, (ICP-MS) for confirmatory analysis. Prior to ICP-MS analysis, 1.0 g of the sample was added to a 50 mL digestion tube. Aliquots of 5 mL deionized water, 2.5 mL concentrated HCl and 2.5 mL concentrated HNO_3_ was added to the tube. The mixture was digested at 95 °C for 2 h in a heating block. The digestate was made to 40 mL with deionized water and centrifuged at 5000* g* for 10 min. The resulting solution was stored at 4 °C and subsequently analyzed with an Agilent 7800 ICP-MS based on US-EPA method 6020B (Amponsah et al., 2022a, 2022b). The limit of detection for the ICP-MS analyses were Ag (< 0.4 mg/kg), As (< 0.3 mg/kg), Cd (0.1 mg/kg), Cr (< 1.6 mg/kg), Mo (< 0.8 mg/kg), Pb (< 0.4 mg/kg), Ni (< 0.8 mg/kg), Sb (< 0.4 mg/kg), Sn (< 1.6 mg/kg) and Zn (< 0.8 mg/kg). The data quality assurance/quality control (QA/QC) program included the analysis of NIST 2711a control samples and duplicates.

**Table 1:** Summary statistics for metal concentrations (mg/kg) in soils

Metal (LOD)	Site	%LOD	Median	Mean	SD	95%tile	Maximum
Ag (< 4)	Community	56	< 3.6	11	13	38	54
	Burning	15	16	20	15	56	56
	Dismantling	0	38	38	6	46	46
As (< 4)	Community	71	< 3.9	5.9	5.1	21	27
	Burning	23	147	191	101	384	384
	Dismantling	40	16	18	8	NA	36
Au (< 4)	Community	73	< 3.6	5.3	5.8	19	31
	Burning	7.7	25	25	10	45	45
	Dismantling	0	28	30	11	47	52
Ba (< 31)	Community	0	401	444	260	1004	1060
	Burning	15	503	481	314	961	961
	Dismantling	0	501	533	171	797	841
Cd (< 6)	Community	48	< 6	15	15	45	79
	Burning	15	52	56	48	175	175
	Dismantling	0	39	38	4.1	42	42
Cr (< 9)	Community	9.7	41	88	98	305	428
	Burning	31	16	139	277	816	816
	Dismantling	0	305	337	94	501	565
Cu (< 15)	Community	0	55	228	1105	219	8730
	Burning	0	20,173	16,996	10,605	30,270	31,297
	Dismantling	0	2847	3257	3012	7831	11,231
Fe (NA)	Community	0	17,509	21,203	19,440	33,480	138,895
	Burning	0	5559	20,120	28,489	75,170	77,048
	Dismantling	0	23,712	32,004	22,494	67,120	93,662
Mn (< 42)	Community	0	270	308	248	578	1939
	Burning	15	118	717	1463	4976	4976
	Dismantling	0	744	884	550	1764	2335
Mo (< 3)	Community	1.6	8.6	9.9	8.6	17	71
	Burning	0	15	18	9.5	34	43
	Dismantling	0	25	24	7.1	34	34
Ni (< 17)	Community	55	< 17	25	15	67	99
	Burning	15	86	138	137	485	485
	Dismantling	0	83	96	65	199	249
Pb (< 4)	Community	1.6	71	122	190	492	1262
	Burning	0	6973	6534	3967	12,200	14,302
	Dismantling	0	755	645	316	983	1015
Pd (< 5)	Community	66	< 4.4	12	14	47	53
	Burning	61	< 4.9	10	10	39	39
	Dismantling	0	47	45	4	50	50
Rb (< 5)	Community	0	29	32	12	53	70
	Burning	0	148	171	78	294	379
	Dismantling	0	58	75	46	162	168
Sb (< 7)	Community	26	13	24	33	72	249
	Burning	7.7	627	554	361	1335	1335
	Dismantling	0	84	105	82	247	257
Sc (< 8)	Community	63	< 7.7	99	161	509	721
	Burning	0	207	312	297	964	1005
	Dismantling	0	611	657	169	926	1094
Se (< 4)	Community	74	< 1.6	2.8	2.5	10	13
	Burning	69	< 3.4	4.9	2.5	12	12
	Dismantling	0	12	13	3.8	18	20
Sn (< 5)	Community	23	10	21	57	50	453
	Burning	7.7	778	1005	975	3151	3151
	Dismantling	0	131	130	79	232	254
Sr (< 7)	Community	0	138	142	81	242	500
	Burning	0	102	103	36	164	175
	Dismantling	0	160	187	74	303	333
Te (< 14)	Community	40	20	29	27	72	184
	Burning	45	< 14	27	15	57	57
	Dismantling	50	< 14	24	12	NA	48
Th (< 5)	Community	24	5.8	11	11	33	47
	Burning	0	31	35	12	51	51
	Dismantling	0	43	45	9.9	57	58
V (< 10)	Community	0	89	101	44	181	251
	Burning	0	44	90	118	238	418
	Dismantling	0	167	174	22	204	206
Zn (< 12)	Community	0	236	514	841	2042	5032
	Burning	0	5224	15,467	27,921	62,580	103,623
	Dismantling	0	1156	1530	1067	3242	3545
Zr (< 7)	Community	0	496	507	130	692	1014
	Burning	7.7	22.6	67	108	335	335
	Dismantling	0	411	386	149	562	590

*LOD* Limit of detection;* %LOD* Percentage of samples with concentrations below the Limit of detection;* StDev* Standard Deviation;* 95%tile* 95 percentile;* NA * Not available

#### In vitro bioaccessibility

The in vitro bioaccessibility assay (IVBA) was based on US-EPA Method 1340 (US-EPA 2007a). A 1 g portion of the sieved soil was extracted by end-to-end rotation for 1 h in 100 mL of 30 g/L glycine adjusted to a pH of 1.5 with concentrated HCl. The extract was filtered and analyzed by ICP-MS as per the methodology used for the total metal analysis. Percent elemental bioaccessibility was calculated for each sample by dividing the concentration in the IVBA extract by the total elemental concentration as determined by acid digestion and ICP-MS analysis. Bioaccessibility was not calculated for the samples with extract concentrations below the limits of detection. The limit of detection for the extracts were Ag (< 1 µg/L), As (< 0.8 µg/L), Cd (0.2 µg/L), Cr (< 4 µg/L), Mo (< 2 µg/L), Pb (< 1 µg/L), Ni (< 2 µg/L), Sb (< 1.0 µg/L), Sn (< 4 µg/L) and Zn (< 2 µg/L). The QA/QC control program included the use of NIST 2711a control samples, procedure and reagent blanks, and duplicates to ensure reproducibility and minimal contamination from filters, vessels, and sample containers.

### Statistical analysis and data evaluation

Statistical analyses were performed using Excel and R software. Since much of the data consisted of results below the limit of detection, Kaplan–Meier statistical techniques for censored environmental data, were used at the 95% confidence limit (Helsel, 2011). In particular, the R package NADA2 (Julian & Helsel, 2022) was utilized for most analyses.

The data was evaluated using international environmental soil quality guidelines since there are currently no Ghanaian soil quality standards. The Canadian Council of Ministers of the Environment (CCME) soil quality guidelines (CCME, 2007) and the Dutch Intervention Values (VROM, 2000) were selected based on their use in other studies conducted in Ghana (Ackah, 2019; Amponsah et al., 2022a, b; Cao et al., 2020). The land use scenario in Agbogbloshie and the surrounding neighborhood included residential, commercial, and industrial and as such the more conservative CCME guideline for residential land use was used for data evaluation.

The potential impact of e-waste on metal concentrations in the receiving environment was evaluated using an enrichment factor while the geoaccumulation index was used to assess the level of pollution (Kowalska et al., 2018). The enrichment factor (EF) was calculated as follows:

1 $$F = \frac{{M_{{\text{s}}} \times {\text{Fe}}_{{\text{b}}} }}{{M_{{\text{b}}} \times {\text{Fe}}_{{\text{s}}} }}$$

where *M*_s_ and Fe_s_ are the concentrations of metal and Fe in the soil sample while *M*_b_ and Fe_b_ are the relevant background concentrations of the metal and Fe. The *M*_b_ for this study was estimated from the mean concentration of the element in 12 control samples collected from Legon Botanical Gardens. An EF < 2 indicates deficiency to minimal enrichment; 2–5 moderate enrichment; 5–20 implies significant enrichment; 20–40 implies very high enrichment; and > 40 extremely high enrichment (Kowalska et al., 2018).

The geoaccumulation index (*I*geo) was calculated using Eq. 2:

2 $$I{\text{geo }} = {\text{ log}}_{{2}} \left[ {\frac{{M_{{\text{s}}} }}{{1.5M_{{\text{b}}} }}} \right]$$

The* I*geo data was evaluated using the following descriptive classes: ≤ 0 = unpolluted; 0–1 = unpolluted to moderately polluted; 1–2 = moderately polluted; 2–3 = moderately to highly polluted; 3–4 = highly polluted; 4–5 = highly to extremely high polluted; and 5–6 extremely high polluted (Kowalska et al., 2018).

### Human health risk assessment

Although workers who dismantle and burn e-waste may be exposed to the soil-borne contaminants through incidental ingestion, the inhalation of smoke and dust particles as well as dermal contact, it is assumed that ingestion is the predominant mode of exposure for the general population. The human health risk assessment (HHRA) was therefore conducted for the oral exposure pathway only incorporating bioaccessibility as a surrogate for oral bioavailability. The risk was estimated using the hazard index (HI) and carcinogenic risk (CR) method. The chemical daily intake (CDI) (mg kg^−1^ day^−1^) was calculated using the Eq. 3 (Health Canada, 2017):

3 $${\text{CDI}} = { }\frac{{{\text{Msoil }} \times {\text{ IngR }} \times {\text{ ET }}}}{{{\text{BW }} \times {\text{ LE}}}}$$

where: Msoil = concentration of metal in soil (mg/kg)

IngR = soil ingestion rate set as 0.0004 kg/day for a child and 0.0001 kg/day for adult (US-EPA, 2007a)

ET = exposure term (unitless) = days/week × weeks/year (× years for carcinogens)

BW = body weight (kg) set as 30 kg for children and 70 kg for adults (WHO, 2021)

LE = life expectancy (years) to be employed for assessments of carcinogens only set as 64 years (World Bank, 2020).

The CDI was divided by the tolerable daily intake (TDI) (mg kg^−1^ d^−1^) and adjusted for relative bioavailability (RBA) to obtain the hazard quotient (HQ) for non-cancer risk (Eq. 4).

4 $${\text{HQ = }}\frac{{{\text{CDI }} \times {\text{RBA}}}}{{{\text{TDI}}}}$$

The RBA for arsenic and lead were determined from the bioaccessibility values (IVBA) using Eqs. (5) and (6), respectively (US-EPA, 2002).

5 $${\text{Arsenic RBA }}\left( \% \right) = 0.{79} \times {\text{ IVBA }}\left( \% \right) + {3}$$

6 $${\text{Lead RBA }}\left( \% \right) = 0.{878 } \times {\text{ IVBA }}\left( \% \right) - {2}.{8}$$

Due to the lack of in vivo/in vitro equations for the remaining metals, their bioaccessibility values were used directly as the surrogate for the RBA.

The TDI values used were Ag: 0.005 mg kg^−1^ d^−1^; As: 0.0003 mg kg^−1^ d^−1^; Cd: 0.0005 mg kg^−1^ d^−1^; Cr: 0.003 mg kg^−1^ d^−1^; Cu: 0.01 mg kg^−1^ d^−1^; Mo: 0.005 mg kg^−1^ d^−1^; Ni; 0.02 mg kg^−1^ d^−1^; Pb: 0.0036 mg kg^−1^ d^−1^; Sb: 0.0005 mg kg^−1^ d^−1^; Sn 0.6 mg kg^−1^ d^−1^; and Zn: 0.3 mg kg^−1^ d^−1^ (Cao et al., 2020; Health Canada, 2008). The HQ for the individual metals (HQi) were summed to obtain the overall hazard index (HI) (Eq. 7).

7 $${\text{HI}} = \sum {\text{HQi}}$$

In this study, the HI represents the sum of the hazard quotients for Ag, As, Cd, Cr, Cu, Mo, Ni, Pb, Sb, Sn, and Zn. HI > 1 implies an elevated probability of non-carcinogenic effects occurring.

Carcinogenic risk (CR) was determined for arsenic only using the Eq. 8:

8 $${\text{CR}} = {\text{CDI }} \times {\text{CSF }} \times {\text{RBA }}$$

where CSF is the cancer slope factor (1.8 mg kg^−1^ d^−1^ for As) (Santé Canada, 2008).

## Results and discussion

### Statistical summary of metals data

Analysis of soils by XRF yielded results for a suite of 33 elements: Ag, As, Au, Ba, Ca, Cd, Co, Cr, Cs, Cu, Fe, Hg, K, Mn, Mo, Ni, Pb, Pd, Rb, S, Sb, Sc, Se, Sn, Sr, Te, Th, Ti, U, V, W, Zn, Zr. The non-metallic element, S, and the major elements (Ca, K, and Ti) were excluded from further analysis. For Co, Hg, U, and W most of the results were below the limit of detection and these metals were also removed from further analysis. Summary Statistics for the XRF soil metal results are presented in Table 1. For censored data, Kaplan–Meier techniques were used at the 95% confidence limit.

To test for a significant difference in means among the three site groupings (Community, Dismantling, and Burning), a pairwise comparison using a Peto and Peto test was carried out for each metal. There were no strong differences by the site for Ba, Ce, Sr, and Te; these metals were removed from further analysis. The remaining 12 toxicologically significant metals were chosen for further treatment and discussion. The elements were evaluated by comparing the mean concentrations for the three site groupings to the CCME guideline for residential land use (CCME, 2007) and the Dutch Intervention values (VROM, 2000). The mean concentrations of the elements As, Cd, Cr, Cu, Mo, Ni, Pb, Sb, and Zn in the Burning and Dismantling areas far exceeded the CCME guideline for residential land use. Except for Cr, the mean concentrations also exceeded the Dutch Intervention values (VROM, 2000).

### Comparison between XRF and ICP-MS data

US-EPA method 6200 requires confirmatory analysis using other techniques such as ICP-MS (US-EPA, 2007a, b). Although previous studies have shown good comparability between the XRF and ICP-MS methodologies used in this study (Amponsah et al., 2022a, b; Darko, Dodd, Nkansah, Ansah, et al., 2017a, b), 13 samples were analyzed using both methods for confirmation. The regression parameters obtained (Table 2) indicated good comparability for the two techniques for most of the metals with *R*^2^ values between 0.62 and 0.99 except for As, Cd, and Cr. The poor comparability for these three elements was probably due to concentration effects, with the XRF yielding relatively high detection limits compared to the ICP-MS. Albeit, the recoveries for the NIST 2711a SRM were Ag (95%), As (96%), Cd (89%), Cr (89%), Cu (83%), Fe (85%), Ni (101%), Pb (93%), Sb (75%), and Zn (91%) which were all within the control limits.

**Table 2:** Regression parameters for X-ray fluorescence and ICP-MS comparability

Element	*R*^2^	Slope	Intercept	*P*-value
As	0.45	3.32	−3.25	0.002
Cd	0.15	0.17	24.9	0.778
Cr	0.21	1.20	1767	0.119
Cu	0.99	0.79	34.1	< 0.001
Fe	0.98	1.50	−3391	< 0.001
Mn	0.89	1.08	188	< 0.001
Mo	0.62	1.35	10.4	0.0015
Pb	0.98	1.07	−3.04	< 0.001
Ni	0.78	0.55	12.7	< 0.001
Sb	0.71	1.05	14.3	< 0.001
Zn	0.83	1.21	−109.6	< 0.001

### Comparison of data to other e-waste sites

The mean elemental concentrations for samples collected from the Burning Area in this study were generally consistent with previous data for Agbogbloshie and other e-waste sites in Ghana and West Africa (Table 3) as well as e-waste sites in Qingyuan and Guiyu, China (Han et al., 2019) and Moradabad, India (Singh et al., 2018). However, Cu, Pb, and Zn concentrations in the Burning Area were higher than any values previously detected. The data thus confirmed the presence of elevated levels of potentially toxic elements at the Agbogbloshie e-waste burning and dismantling areas.

**Table 3:** Comparison of select mean metal concentrations (mg/kg) in soil from different e-waste sites and international soil quality guidelines

Location	As	Cd	Cr	Cu	Ni	Pb	Sb	Sn	Zn	Sampling Time	Reference
Dismantling Area	19	38	337	3257	96	645	105	130	1530	Dec 2020	This study
Burning Area	191	56	139	16,700	138	6534	554	1005	15,470	Dec 2020	This study
Dagomba Line Ghana^D,B^	80	7.2	116	2608	79	1273	151		1714	Feb 2018	(Amponsah et al., 2022a, b)
Agbogbloshie, Ghana^B^	271	11	103	11,200	NA	2380	NA	585	1820	Dec 2016–Jan 2017	(Ackah, 2019)
Agbogbloshie, Ghana^D^	8	14	139	10,400	NA	5080	NA	1130	7010	Dec 2016–Jan 2017	(Ackah, 2019)
Agbogbloshie, Ghana	12	11	NA	6590	NA	4540	687	NA	NA	NA	(Cao et al., 2020)
Ashaiman, Ghana	6	4	59	6060	NA	1690	NA	599	2010	Dec 2016–Jan 2017	(Ackah, 2019)
Ibadan, Nigeria	NA	2.5	42	3483	24	5650	NA	NA	NA	NA	(Adesokan et al., 2016)
Makea, Duola, Cameroon	NA	20	130	130	56	290	NA	NA	160	Feb–Jun 2017	(Ouabo et al., 2019)
Ngodi, Duola, Cameroon	NA	30	70	80	50	310	NA	NA	150	Feb–Jun 2017	(Ouabo et al., 2019)
New Bell, Duola, Cameroon	NA	20	70	80	53	280	NA	NA	155	Feb–Jun 2017	(Ouabo et al., 2019)
CCME R/P Guideline	12	10	64	63	45	140	20	50	250		(CCME, 2007)
Dutch Intervention	55	12	380	190	210	210	15	NA	720		(VROM, 2000)

*B* Burning area, *D* Dismantling area, *NA* not available

### Pollution indices

**Fig. 2:**
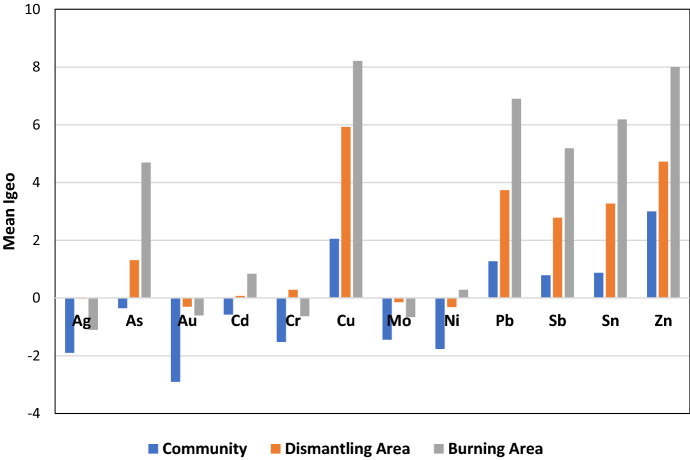
Mean *I*geo for elements in the Community, Dismantling, and Burning Areas

**Fig. 3:**
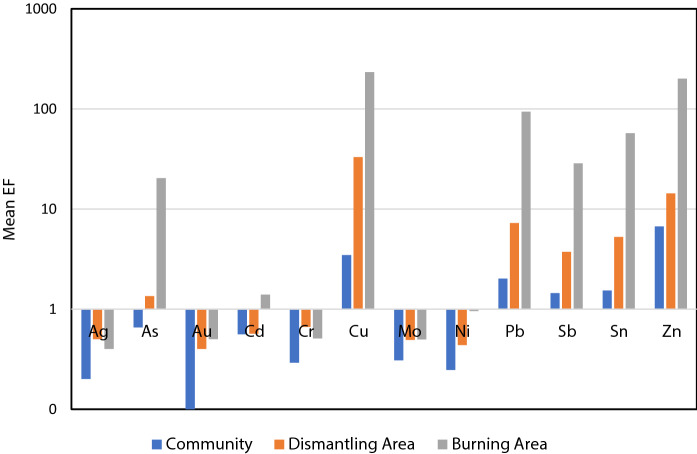
Mean Enrichment Factor (EF) for select elements in the Community, Dismantling, and Burning Areas at Agbogbloshie

The *I*geo values (Fig. 2) corroborated the presence of the elevated levels of metals at both the dismantling area and the Burning Area with the soils at the Burning Area showing high to extreme pollution with As, Cu, Pb, Sb, Sn, and Zn. This observation was also confirmed by extremely high enrichment factors for these elements (Fig. 3).

### Elemental distribution at Agbogbloshie and the surrounding community

**Fig. 4:**
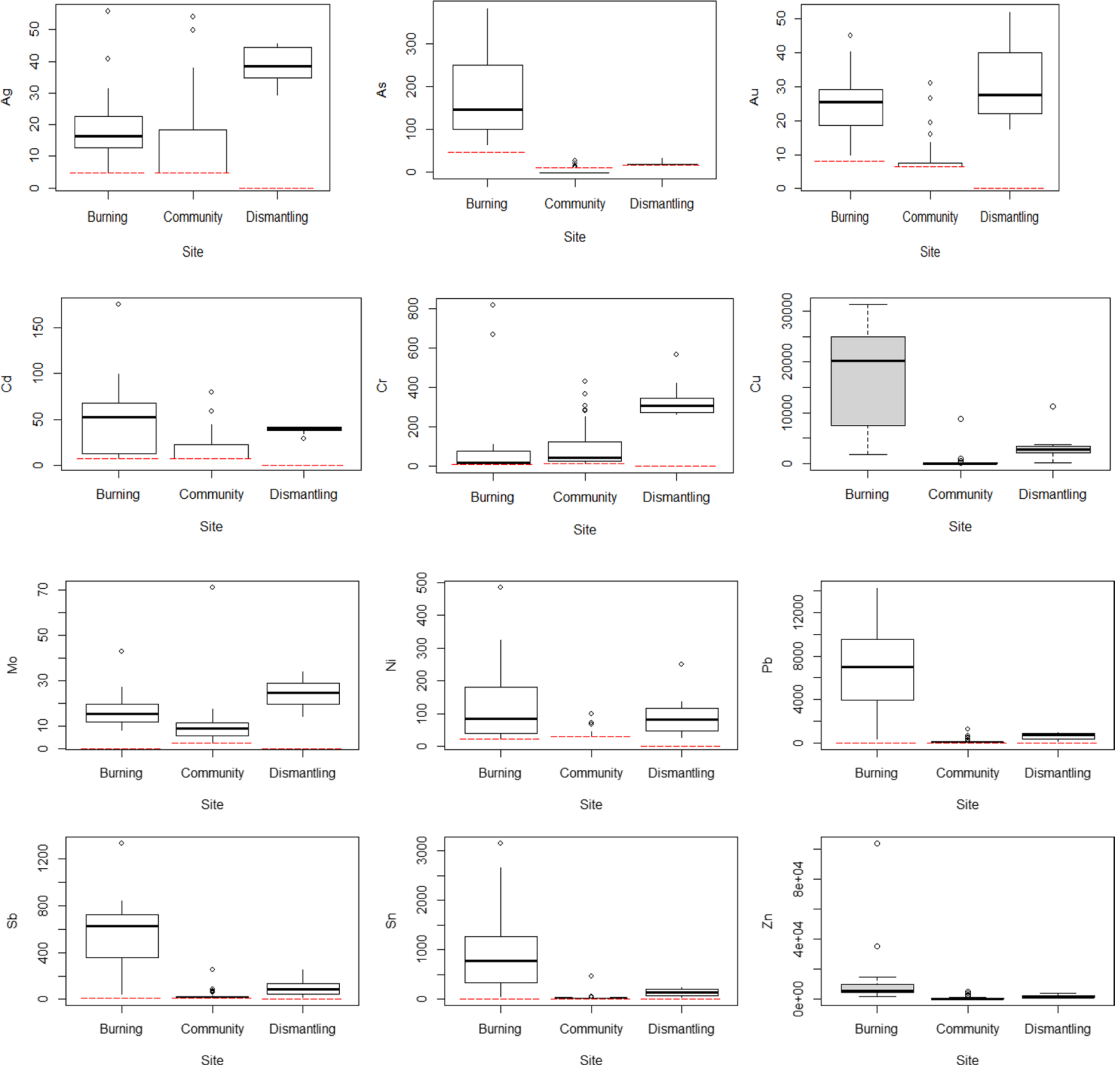
Boxplots of elemental concentrations (mg/kg) at the Burning, Dismantling, and Surrounding Community at Agbogbloshie. For boxplots including censored data the detection limit is given by the dashed line and the boxes are not shaded

Elemental distribution at the Burning, Dismantling, and Community were explored further through boxplots (Fig. 4). The median concentrations for Ag, Au, and Mo followed the order Community < Burning < Dismantling suggesting the Dismantling area may be introducing these contaminants into the environment. On the other hand, the median concentrations of Cd, Cu, Ni, Pb, Sb, Sn, and Zn were relatively higher in the Burning Area and followed the order Community < Dismantling < Burning. It appears the burning of e-waste and other debris contributed to the increase of these elements in the environment. The distribution of Cr was different in that the median concentrations followed the order Burning < Community < Dismantling.

Despite the significant differences between concentrations for these elements in the community vs the burning and dismantling areas, there were no strong correlations with the distance of the community sampling site from the burning or dismantling sites. Weak correlations were observed for Cu (*p* = 0.04, distance to burning site; *p* = 0.05 distance to dismantling site), Ni (*p* = 0.03, distance to dismantling site), Sb (*p* = 0.04, distance to dismantling site), and As (*p* = 0.03, distance to burning site) lending some support for off-site migration to the community from the recycling areas.

**Fig. 5:**
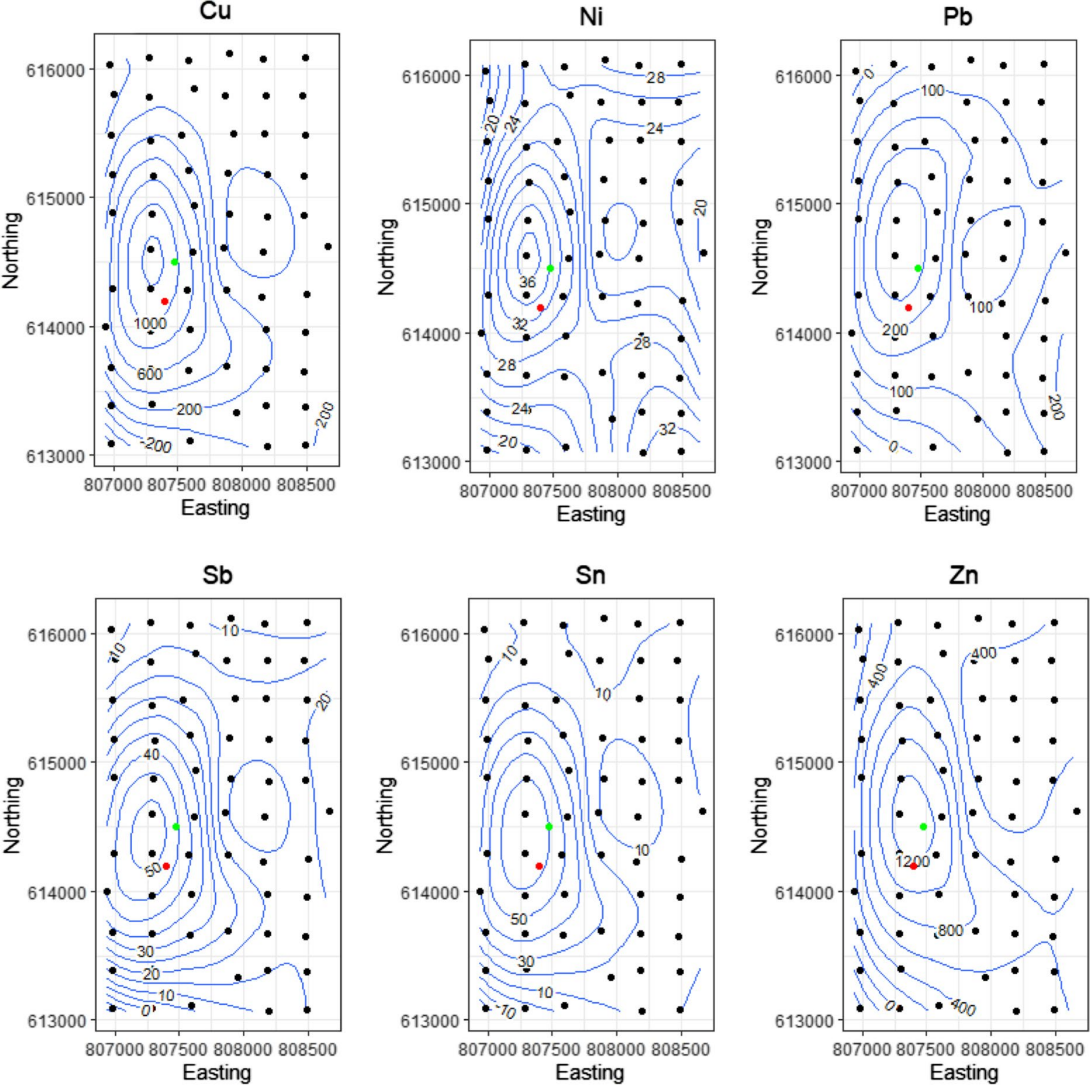
Contour Plots of Cu, Ni, Pb, Sb, Sn, and Zn in the Community samples showing elevated metal concentrations (mg/kg) closer to Burning (red dot) and Dismantling Area (green dot)

Contour plots of the concentrations of Cu, Ni, Pb, Sb, Sn, and Zn showed clear increases in concentration closer to the burning and dismantling sites (Fig. 5). These observations corroborate the suggestion that activities at the e-waste recycling area may be contributing to elevated elemental levels in the surrounding community.

### Principal component analysis and correlation between elements

**Fig. 6:**
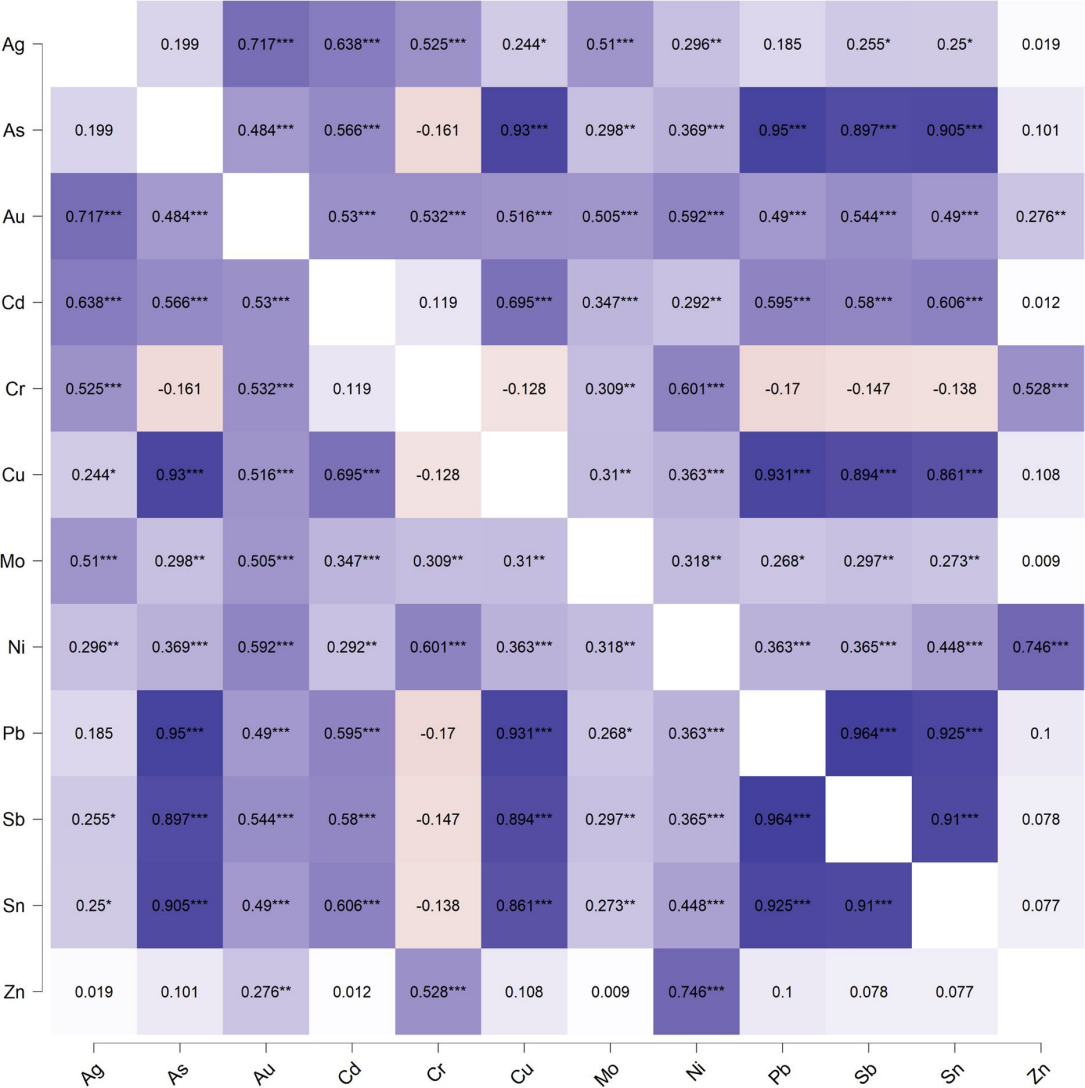
Pearson’s heat map showing inter-element relationships. Note: *p < 0.05, **p < 0.01, ***p < 0.001

The relationships between the elements were explored using correlation analysis. Except for Cr, there was a high positive correlation among the elements at Agbogbloshie as shown in the heat map in Fig. 6. For instance, As correlated highly with Cu, Pb, Sb, and Sn with coefficients of 0.93, 0.95, 0.897, and 0.905 (*p* < 0.001), respectively. The high positive and significant correlations between the elements suggest that they accumulated from similar sources (i.e., dismantling and burning of e-waste and other materials at Agbogbloshie). Although the relationships were not statistically significant, Cr concentration decreased as As, Cu, Pb, Sb, and Sn increased in the soil at Agbogbloshie (i.e., Cr contamination may originate from a different source).

Principal component analysis was performed to further explore the sources of elemental concentration (Table 4). Eigenvalues > 1 were only considered for interpreting the results. Three principal components (PC) were identified which collectively accounted for 85.2% of the overall variation using varimax rotation. The PC1 constituted by As, Cd, Cu, Pb, Sb, and Sn explained 50.8% of the overall variance, and PC2 constituted by Ag, Au, Cd, Cr, and Mo contributed 22.0% of the total variance. Chromium, Ni and Zn constituted PC3 and accounted for 12.4% of the variance. The PC1 indicated metals are sourced from the same activity (the burning of e-waste) while the metals associated with PC2 were attributed to the dismantling activities. The metals associated with PC3 (Cr, Ni, and Zn) may be attributed to natural enrichment and other industrial activities such as scrap metal dealerships in the vicinities of the Agbogbloshie e-waste recycling site. The considerably high degree of contamination, coupled with the tendency for contaminants to be spatially autocorrelated and the random variation in contamination factors all suggest that the pollution is caused by anthropogenic activity, i.e., the e-waste recycling activity.

**Table 4:** Component loadings for the principal component analysis of select elements at Agbogbloshie and the Surrounding Area

Element	PC1	PC2	PC3	Uniqueness
Ag		0.932		0.121
As	0.953			0.070
Au		0.718		0.193
Cd	0.572	0.599		0.312
Cr		0.562	0.685	0.116
Cu	0.938			0.077
Mo		0.699		0.477
Ni			0.861	0.103
Pb	0.975			0.031
Sb	0.943			0.076
Sn	0.934			0.093
Zn			0.939	0.109

*Note.* Applied rotation method is varimax

### Bioaccessibility and human health risk assessment

The statistical summary for As, Cr, Cu, Pb, Ni, and Zn in vitro bioaccessibility is presented in Table 5. Bioaccessibility was not calculated for the remaining metals since their concentrations in most of the extracts were below the limits of detection. Bioaccessibility was variable among the metals with mean values in the order Pb > Cu > Zn > Ni > As > Cr. This order is similar to that obtained for soil samples collected from an e-waste site recycling site at Dagomba Line, Ghana (Amponsah et al., 2022a, 2022b). Lead bioaccessibility which varied from 51.3 to 91.9% was comparable to the range of 49.0–90.2% reported for Dagomba Line but higher than the 6.7–56% previously reported for the gastric phase bioaccessibility for e-waste burning sites in Agbogbloshie (Cao et al., 2020). Arsenic bioaccessibility values (12.4–41.6%) were also comparable to the values obtained for Dagomba Line (4.1–21.1) and Agbogbloshie (2.1–39.8%). The As and Pb bioaccessibility values were adjusted for the RBA using their respective in vivo/in vitro regression equations (Eqs. 5 and 6) and subsequently used for calculating the hazard quotients (Eq. 4). The bioaccessibility values of Ag, Cr, Cu, Ni, and Zn were used directly due to the lack of suitable in vivo/in vitro equations. For the remaining elements (Cd, Mo, Sb, and Sn) whose bioaccessibility values were not calculated due to the concentrations in the extracts being below detection, an RBA of 1 was used in Eq. 4.

**Table 5:** Summary statistics for metal bioaccessibility (%)

	Ag	As	Co	Cr	Cu	Pb	Ni	Zn
Mean	65.8	23.3	41.3	8.8	58.9	67.5	38.3	58.4
SD	29.7	9.7	12.6	4.8	12.2	12.0	6.0	20.2
Median	58.6	17.8	43.0	8.0	63.8	66.1	37.8	66.1
Minimum	40.8	12.4	14.6	2.2	39.5	51.3	26.4	13.9
Maximum	145.2	41.6	58.7	16.9	80.9	91.1	45.4	88.3
95%tile	131.5	38.9	56.4	16.7	73.7	86.3	45.2	76.6
Variance	882.6	94.1	158.3	22.9	149.1	143.6	36.5	407.7

**Fig. 7:**
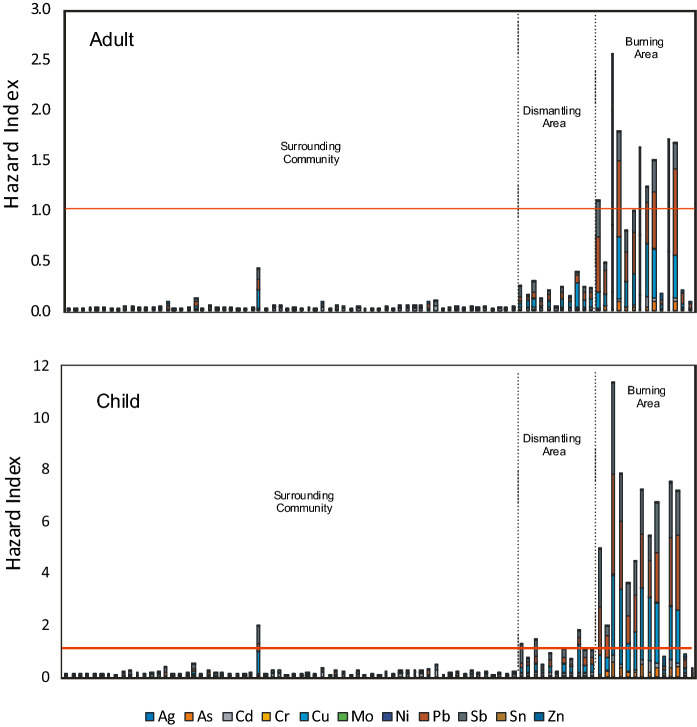
Hazard indices for metals in soils at Agbogbloshie and the Surrounding Area Samples above the red line show unacceptable noncarcinogenic risk

The non-carcinogenic hazard indices for adults and children receptors represent the sum of the hazard quotients for Ag, As, Cd, Cr, Cu, Mo, Ni, Pb, Sb, Sn, and Zn for each of the sampling locations are summarized in Fig. 7. For adults, unacceptable levels of non-carcinogenic effects (HI > 1) were noted for 8 of the 14 samples collected from the Burning Area while all the samples collected from the Dismantling Area and the Surrounding Community had HI below 1. The non-carcinogenic risk for children was much higher with 10 samples from the burning area, 6 samples from the Dismantling Area and 1 sample from the Surrounding Community with HI > 1. The single sample in the Surrounding Community that had a high HI was collected near a Mechanic/Fuel Station; the elevated risk may be associated with activities at this point source. As with previous investigations at Agbogbloshie and other e-waste sites (Amponsah et al., 2022a, b; Cao et al., 2020; Leung et al., 2008), Pb and Cu were the leading contributors to the elevated human health risk via oral ingestion.

**Fig. 8:**
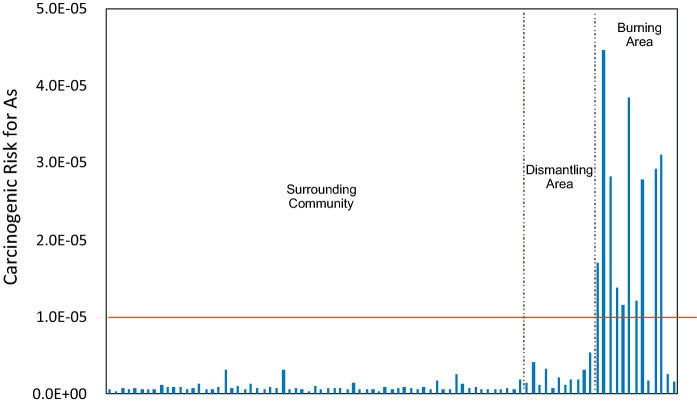
Carcinogenic risk for arsenic in soil samples at Agbogbloshie and the surrounding area. Samples above the red line show unacceptable risk

The cancer risk for As for adults was greater than 1 × 10^−5^ at 11 out of the 14 sites as shown in Fig. 8. This represented an unacceptable risk based on Canadian guidelines that the acceptable risk is one excess cancer death per 100,000 people exposed (Health Canada, 2004). All samples from the Surrounding Community and the Dismantling area had As cancer risk that was lower than 1 × 10^–6^ indicating that there were no carcinogenic adverse health risks for adults.

## Conclusion

The distribution, bioaccessibility, and potential health risks associated with toxic metals in surface soils of Agbogbloshie and its surrounding communities were determined. Agbogbloshie is a hotspot for informal e-waste recycling activities, thus, concentrations of metals such as As, Cd, Cr, Cu, Mo, Ni, Pb, Sb, Sn, and Zn were found to exceed international environmental soil quality guidelines. Although lower concentrations were recorded in the surrounding communities, concentrations of Cd, Cr, Cu, Sb, and Zn at certain locations exceeded soil quality guidelines for residential land use. The metals including Ag, Cu, Pb, Ni, and Zn were highly bioaccessible. Even though bioaccessibility was considered in calculating human health risks, non-carcinogenic, and cancer risks were found at unacceptable levels in the e-waste recycling site. Results from this study show that human activities at the Agbogbloshie recycling site could potentially lead to adverse human health and ecological effects in the surrounding communities.

## Data Availability

All data generated or analyzed during this study are included in this published article.
